# Osteoclasts and CD8 T Cells Form a Negative Feedback Loop That Contributes to Homeostasis of Both the Skeletal and Immune Systems

**DOI:** 10.1155/2013/429373

**Published:** 2013-06-09

**Authors:** Zachary S. Buchwald, Rajeev Aurora

**Affiliations:** Department of Molecular Microbiology and Immunology, Saint Louis University School of Medicine, 1100 S. Grand Boulevond DRC 605, St. Louis, MO 63104, USA

## Abstract

There are a number of dynamic regulatory loops that maintain homeostasis of the immune and skeletal systems. In this review, we highlight a number of these regulatory interactions that contribute to maintaining homeostasis. In addition, we review data on a negative regulatory feedback loop between osteoclasts and CD8 T cells that contributes to homeostasis of both the skeletal and immune systems.

## 1. Introduction

Osteoimmunology is the study of the crosstalk between the skeletal and immune systems. The term osteoimmunology emerged from the recognition [1] that many lymphocyte-derived cytokines including interleukin (IL)-17, type I and II interferons, and RANKL are potent mediators of osteoclast function and differentiation [2–4]. Effector T-cell-produced proinflammatory cytokines have been shown to promote bone erosion in inflammatory diseases such as rheumatoid arthritis and periodontitis and to also play a critical role in bone cancers and postmenopausal osteoporosis [5–7]. In this review, we discuss some of the principles of design of the regulatory interactions that maintain homeostasis for both the immune (first one-third of the review) and skeletal (second third) systems. Finally, we discuss our studies on a new negative regulatory feedback loop we discovered between osteoclasts and CD8 T cells in the context of these homeostatic regulatory interactions.

### 1.1. Homeostasis

All self-assembling, self-regulating systems need to maintain an internal stable state (i.e., a set point) in response to external changes, stimuli, or inputs. The regulatory mechanisms that act to maintain or restore the stable state are homeostatic regulators. Both the immune and skeletal systems are highly regulated by layers of hierarchical networks of cellular interactions to maintain stability and provide a balanced response to physiological and environmental changes. The immune system and the skeletal system require positive and negative regulators to maintain homeostasis. 

## 2. Self-Regulation in the Immune System

**Figure 1:**
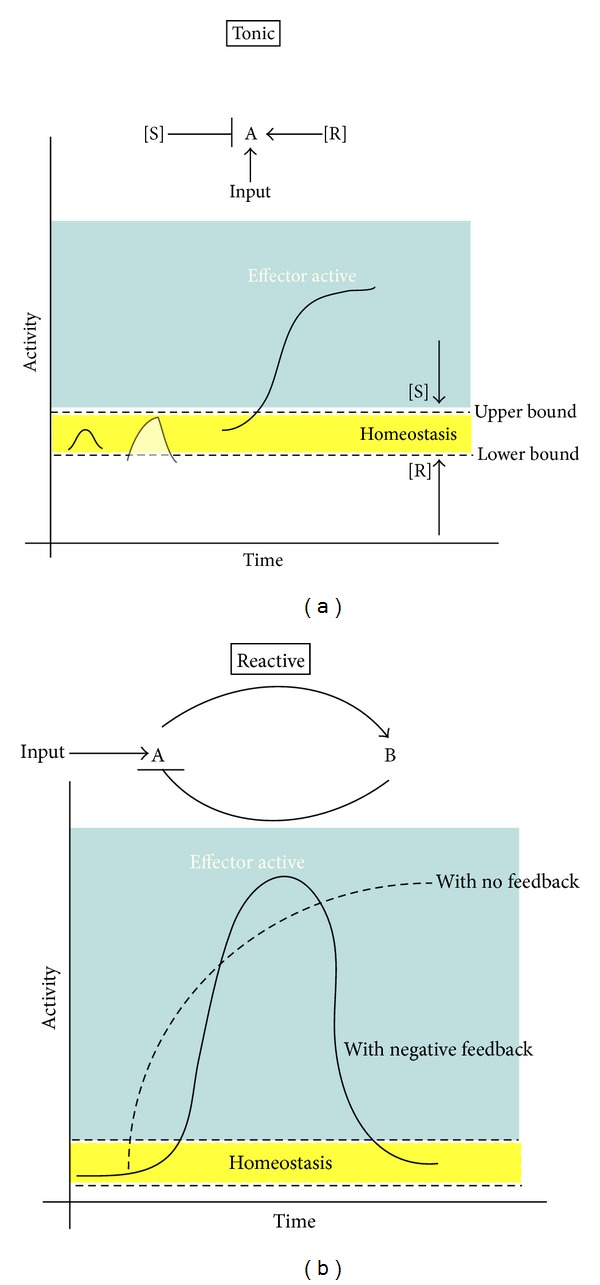
Tonic and reactive regulatory motifs: (a) the tonic regulatory motif maintains homeostasis by adjusting the concentration of a suppressor [S], which sets the upper bound of the activating threshold. In some cases, basal levels of an activator [R] establish the lower bound. (b) The reactive regulatory motif maintains homeostasis typically through a negative feedback loop. Negative feedback produces spiked activation and oscillations under some circumstances.

The central feature of the immune system is to distinguish self from nonself and to mount a response to non-self. However, as has been previously noted [8, 9], the problem is more complex. Because of the random nature by which the B-cell and T-cell repertoire is generated and because of the limits of central tolerance, there is a constant risk of antiself-responses by cells of the adaptive immune system. In studying the mechanisms that *suppress* the immune system, three principles of design have emerged. First, there are recognizable patterns in structures of regulatory pathways (subnetworks) that operate within a cell (molecular), at the level of cell-cell interactions and at a system-wide level. While the specific mediators may vary at each level, the overall architecture of these subnetworks is conserved to form recognizable motifs [10, 11]. Second, analysis of the motifs revealed two classes of regulatory subnetworks: tonic and reactive. Tonic regulators set the threshold above which the stimulus (or Input in Figure 1(a)) must rise to elicit a response; they prevent activation from happening. An example of a tonic regulator is TGF*β*1, that is present in an active form in many tissues. TGF*β*1 raises the functional activation threshold for lymphocytes. By tweaking the concentration of the suppressor, inappropriate weak responses are suppressed (Figure 1(a)). Reactive regulators control the emerging response; they limit the intensity once it has begun [12] (Figure 1(b)). An example of a reactive regulator is IL-10 which is produced by the innate and adaptive immune arms, and which limits immune response and inflammation [13]. Microbial products (Input in Figure 1(b)) binding to Toll-like receptor (TLR) in myeloid dendritic cells (A in Figure 1(b)), for instance, leads to maturation of dendritic cells and activation of T cells (B in Figure 1(b)). Activated T cells produce IL-10, which acts on the dendritic cells, to limit the subsequent activation of T cells [14]. The response to all cytokines is context dependent, and therefore these examples are for illustration and not intended as a blanket rule. For example, while TGF*β* is a tonic regulator of the T cell response, in combination with IL-6 it induces a highly pathogenic (proinflammatory) T_H_17 response [15]. The third emerging principle of design is the spatiotemporal negative regulation. The immune response is a process, or a sequence of coordinated events, with an initiation, maintenance, and resolution phase. Therefore, the regulatory kinetics must be reactive and lead to a restoration of homeostasis. This means that there is a time delay between the initiation phase and full activation of the resolution phase. The resolution phase initiates the shutdown of the immune response, to prevent excess tissue damage, and initiates wound healing and repair. For instance, Toll-like receptor signaling, which sense, and triggers responses to pathogen-associated molecular patterns (PAMPS), is regulated at multiple levels. Lang and Mansell conclude that “*the negative regulators of TLR signaling do not work as a single entity, but rather akin to an orchestral score, each component is reliant on its other instruments to produce a melody rather than a crashing cacophony*” [16]. 

Regulatory T cells, another type of immunologic suppressor, are more difficult to classify. T cells that express CD25 and FoxP3 are known as regulatory T cells (T_REG_). The best-studied T_REG_ are CD4^+^ CD25^+^ FoxP3^+^ that work in both the tonic and reactive modes. Depletion of T_REG_, either at the cellular level or by genetic lesion of FoxP3, leads to multiorgan autoimmune disease [17–22]. There is direct evidence that they work mechanistically in a tonic mode to suppress activation of self-reactive T cells [23]. Germain et al. [24] have elegantly argued that T_REG_ primarily work in a reactive mode as they often act after initial activation in response to nonself-antigens to suppress the development [25] and/or function [26] of effector T cells. 

## 3. Regulatory Mechanisms That Maintain Homeostasis in the Skeletal System

Bone is remodeled thoughout life. Skeletal system homeostasis is maintained primarily by osteoclasts, which resorb bone, and osteoblasts, which generate new bone. Bone remodeling is carried out in spatially discrete foci by a team of cells that form a basic multicellular unit (BMU) or a bone-remodeling compartment (BRC) for cancellous bone. The formation of the BMU occurs as a sequence of events: origination, osteoclast recruitment, resorption, osteoblast recruitment, osteoid formation, and finally mineralization [27]. Many of the principles of design of regulatory networks observed in immune system, like hierarchical, tonic versus reactive, and spatiotemporal regulation, also appear in the skeletal system for maintaining and restoring homeostasis. There are local interactions for each step of the remodeling process: osteocytes embedded in bone, produce receptor activator of NF-*κ*B ligand (RANKL) that is needed for the differentiation and activity of osteoclasts [28, 29]. Osteocytes transduce mechanical forces in accordance with Wolff's law to regulate osteoclast activity through production of RANKL [30] ([R] in Figure 1(a)). Osteoblasts also produce a decoy receptor of RANKL, osteoprotegerin (OPG), which plays an important role in suppressing ([S] in Figure 1(a)) osteoclast activity by blocking RANKL binding to its receptor. These interactions are best characterized as tonic regulators as they set the threshold at which remodeling activity of the BMU begins. 

**Figure 2:**
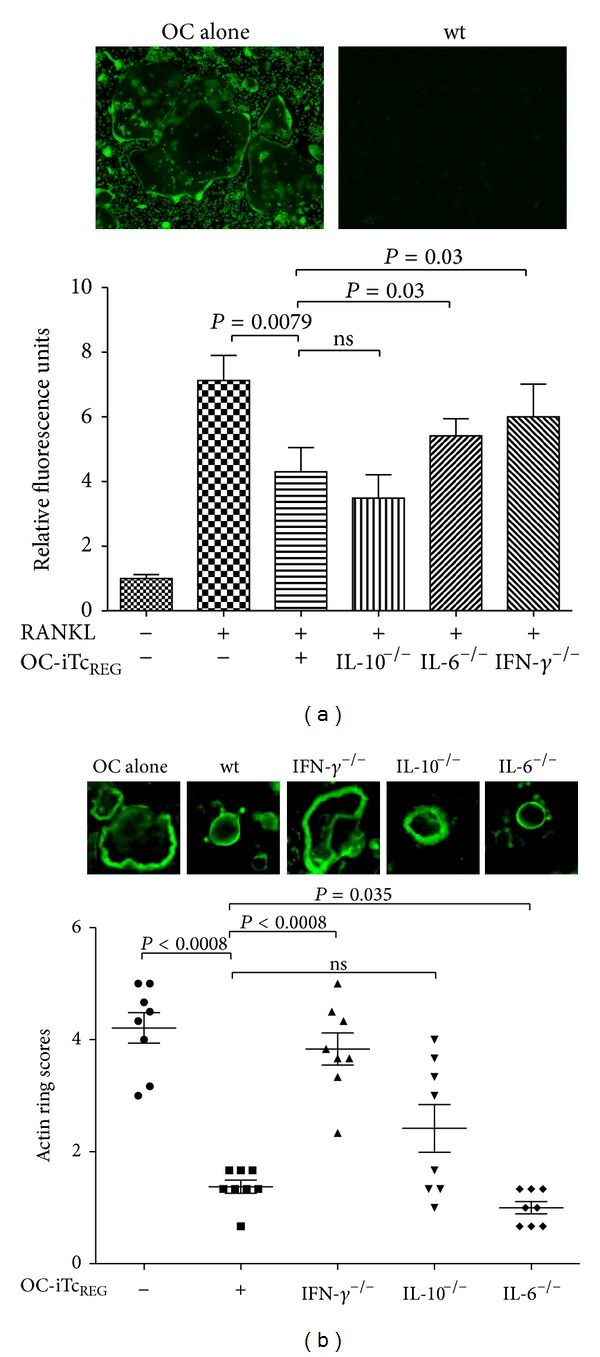
OC-iTc_REG_ suppress osteoclast differentiation and actin ring formation: (a) OC-iTc_REG_ were generated from polyclonal wt, IL-10^−/−^, IL-6^−/−^, or IFN-*γ* ^−/−^ splenic T cells and cocultured with osteoclast precursors in the presence of RANKL and M-CSF for 5 days. The T cells were then removed and the adherent cells were assayed for TRAP activity using a fluorescent substrate ELF97. IFN-*γ* and IL-6 were found to be necessary for OC-iTc_REG_ anti-osteoclastogenic activity. (b) Osteoclasts are differentiated on bovine bone slices and then cultured for 24 hrs with wt, IL-10^−/−^, IL-6^−/−^, or IFN-*γ* ^−/−^ OC-iTc_REG_. The T cells were then removed and the osteoclasts were stained with fluorophore-conjugated phalloidin to assay for actin ring formation. IFN-*γ* was shown to be required for the OC-iTc_REG_ to suppress actin ring formation.

At homeostasis, bone resorption rate is “coupled” or balanced by bone formation rate at the organismal level and within the BMU. To maintain the balance, osteoclasts express membrane-bound Ephrin (Eph) B1 and B2 which regulates the differentiation of the osteoblast through its interaction with EphB4 [31], and hence maintains balance in the two rates. In addition to EphB2, secreted factors (i.e., S1P and MCP-1) keep the osteoclast and osteoblast activity closely coupled (see Figure 2). These interactions maintain bone mass at a set point and therefore are examples of tonic regulation. 

Bone is major store of calcium and phosphate. A number of endocrinal or bone extrinsic factors regulate the BMU to release or reduce these minerals from serum. These regulators are typically reactive, as they can initiate or suppress resorption and bone formation in response to changes in serum calcium and phosphate levels. The kidney and parathyroid sense levels of circulating calcium and phosphate and produce hormones. For instance, low calcium levels lead to increased parathyroid hormone (PTH) levels to stimulate bone resorption and the release of calcium into the serum. These sensors and hormonal regulatory “wires” also form subnetworks within a hierarchical network to regulate bone homeostasis. Another example is the endocrine subnetwork formed between osteoblasts and adipocytes through adiponectin that regulates fat storage and bone mineral density. In summary, while there are specific differences between the immune and skeletal system, they can be both described by similar principles of design. 

### 3.1. Immune Regulation of Bone Homeostasis

The immune system produces mediators that can alter tonic regulation of bone homeostasis. Some activated proinflammatory T cells express RANKL [32] that leads to bone erosion [33]. It was also recognized that activated T cells, in some cases, express interferon-*γ*, which mitigates the proresorptive effect of RANKL by promoting the degradation of the receptor associated factor TRAF6 through which RANKL signals [34]. The dynamics of pro- and antiresorptive effects on osteoclasts by T cells led Arron and Choi to coin the term osteoimmunology [1]. Further discoveries that other cytokines and other mediators produced by activated T cells and tumors can lead to increased expression of RANKL, and hence to bone loss, also led development of Denosomub, a humanized antibody that blocks RANKL [13]. As described above, RANKL and OPG maintain the upper and lower bounds of regulating osteoclast function acting as tonic regulators. The expression of both these proteins has been ascribed to osteoblasts and osteocytes [28, 29]. Li et al. have shown that B cells are also a significant source (up to 45% of total bone marrow production) of OPG when stimulated through CD40 [35]. Consistent with the notion that activation of B cells requires T cell helper functions (notably IL-4 secreting T_H_2 subset), the CD40 ligand (CD154) is expressed on activated T cells (reviewed in [36]). Therefore, the field of osteoimmunology has focused to date on the regulation of bone homeostasis by the immune system through modulating tonic regulators. 

## 4. A Negative Feedback Loop between Osteoclasts and CD8 T Cells

In this section, we describe results from our laboratory that provide a new fundamental link between the immune and skeletal systems. Genetic studies have identified many regulatory proteins that control the development of osteoclasts, but the relationships between these genes and the processes they regulate have not been well understood. To that end, we performed a time course microarray looking at osteoclast differentiation from bone-marrow-derived precursor cells [37]. We observed an upregulation of genes for many proteases, the machinery required for transcytosis, protein processing, protein sorting, the MHC class I subunits, and T-cell chemoattractants CXCL10 and CCL5. These observations suggested that osteoclasts possess T-cell recruitment and priming activity.

Using C57BL/6 splenocytes, we demonstrated that osteoclasts selectively recruit CD8 T-cells *in vitro* [38]. Additionally, osteoclasts can endocytose extracellular antigen, process full-length protein in a proteasome-dependent manner, cross-present that antigen on MHC-I, and activate antigen specific CD8 T cells. Li et al. have also shown activation of CD8 T cells by human osteoclasts that were derived from peripheral blood mononuclear cells [39]. The murine osteoclast-activated CD8 T cells were shown to be noncytolytic and anergic. They express CD25 and FoxP3, and therefore we refer to them as osteoclast-induced regulatory CD8 T cells or OC-iTc_REG_. Further characterization of these cells revealed that they express membrane-bound RANKL, CTLA-4 and produce IFN-*γ*, IL-6, IL-10, and IL-2 [38, 40]. Interestingly, while RANKL promotes osteoclast differentiation, IFN-*γ* and IL-10 are known negative regulators of osteoclastogenesis. As these Tc_REG_ express positive and negative regulators of OC, we tested to see what the net effect of  Tc_REG_ is on osteoclasts *in vitro*. Using a cocultured OC-iTc_REG_ and mature osteoclasts, we demonstrated that OC-iTc_REG_ suppress osteoclast activity *in vitro*. Indeed, OC-iTc_REG_ suppressed osteoclastogenesis from precursor cells and the actin ring formation in mature osteoclasts [40]. Using T cells from mice with targeted knock out of IL-6, IL-10, and IFN-*γ* we found that loss of IFN-*γ* or IL-6 restored osteoclastogenesis, whereas loss of IFN-*γ* (and IL-10 weakly) restored actin ring formation (Figure 2). 

To determine the ability of the OC-iTc_REG_ to suppress bone turnover *in vivo*, we used two different models: first, we used RANKL administration to activate osteoclast activity and, using a series of adoptive transfer experiments, we showed that OC-iTc_REG_ have the ability to limit bone turnover *in vivo *[41]. Using CD8 T cells from rescued Scurfy mice, which cannot express functional FoxP3, we showed that FoxP3 expression is required for the ability of CD8 T cells to limit bone turnover. As a second model to test the ability of *ex vivo*-generated OC-iTc_REG_ to limit bone loss, we used ovariectomized mice, which have increased turnover because of the loss of estrogen. These experiments demonstrated that the OC-iTc_REG_ could limit bone loss to a similar level as the bisphosphonate, Zoledronate. Furthermore, treatment with OC-iTc_REG_ allowed for bone repair, as both the bone formation rate (BFR) and mineral apposition rate (MAR) increased relative to Zoledronate-treated mice [41]. Since OC-iTc_REG_ could inhibit osteoclastogenesis *in vitro*, we tested whether OC-iTc_REG_ could reduce osteoclast numbers in ovariectomized mice. Osteoclast numbers were quantified using serum TRAP5b and via bone histomorphometry. We observed a statistically significant drop in osteoclast numbers in treated mice compared to control ovariectomized mice [41]. Finally, we also found that ovariectomized mice had increased levels of effector T cells (CD3^+^ and CD44^+^) relative to sham-operated mice; treatment with OC-iTc_REG_ decreases the fraction of effector T cells to levels observed in sham-operated mice. The latter results demonstrate that OC-iTc_REG_ have regulatory T cell activity *in vivo*. 

**Figure 3:**
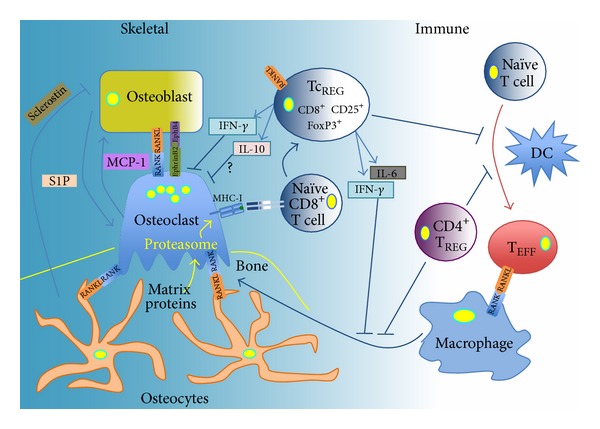
Regulatory network between the bone and immune system: the figure shows the network architecture of regulatory interactions that maintain homeostasis of the skeletal systems. Osteoclast-induced FoxP3^+^ CD8 T cells are capable of regulating the skeletal and the immune systems.

At the same time as the discovery that effector T cells (produced under proinflammatory conditions) promote bone turnover, by secreting RANKL and type I and type II interferons, experiments showed that T cells, CD8 T cells in particular, were protective against bone turnover [42, 43]. For instance, it was noted that when bone marrow cells from TCR*α* ^−/−^ mice, which lack CD4 and CD8 T cells, were cultured in the presence of 1,25(OH)_2_, vitamin D3 osteoclastogenesis was enhanced indicating that T cells suppress osteoclastogenesis [44]. Our findings provide a mechanism for the role of the FoxP3^+^ subset of CD8 T cells in regulating osteoclastogenesis (Figure 3). Zaiss et al. have shown that CD4 T_REG_ can also suppress bone turnover in both osteoporosis and rheumatoid arthritis models [45–47]. 

## 5. The Physiological Roles of Tc_**REG**_

Tc_REG_ have been documented in humans and mice [48–59], but they have not been studied extensively, in part due to their low abundance (0.2 to 2% of CD8^+^ T cells) in lymphoid organs. In comparison, the well-studied CD4^+^ regulatory T-cells, T_REG_, comprise 5–12% of CD4^+^ T-cell in the spleen. However, the low abundance of a regulator does not belie its importance. Indeed, most regulators are present in low abundance. For instance, transcription factors, present at <0.1% of cellular proteins [60] and whose concentration is exquisitely regulated, are critical regulators of gene expression [61].

Tc_REG_ and T_REG_ have overlapping and distinct functions. Both cells express the transcription factor, FoxP3 a marker of regulatory T cells [21, 62, 63]. The two regulatory T cells are controlled differently: thymically and peripherally produced T_REG_ require restimulation through their T-cell receptor (TCR) by MHC class II to express their suppressive effector functions [64]. The maturation of antigen presenting cells (APC) that express MHC class II, needed for restimulation, is tightly regulated [65, 66]. In contrast, Tc_REG_ do not require restimulation [40]. In any case, as all cells (except RBC) constitutively express MHC class I, any cell could potentially stimulate Tc_REG_. Our studies [40] and others [67–69] have shown that Tc_REG_ are regulated by induction locally (e.g., in the bone marrow) from naïve CD8 T-cells; hence, their steady state abundance would be low in lymphoid tissue. 

The ability of osteoclasts to induce Tc_REG_ and the ability of Tc_REG_ to subsequently regulate osteoclast function establish a bidirectional regulatory loop between these two cells in the bone marrow. Notably, the regulatory loop does not require the presence of proinflammatory cytokines. Indeed, our ability to isolate functional Tc_REG_ from mice, in the absence of any inflammatory disease [40], indicates that these cells have a role in maintaining skeletal homeostasis *in vivo*. In contrast to CD4 T_REG_, the Tc_REG_ may have a more specialized and local function. The ability of osteoclasts to induce Tc_REG_ and their ability to suppress without restimulation may provide an explanation for the low levels of Tc_REG_ found *in vivo*: the system is rapidly inducible, so a large reservoir of Tc_REG_ is not needed. The induction of the Tc_REG_ by osteoclasts that suppress osteoclasts would be self-limiting and lead to small number of Tc_REG_. This line of reasoning indicates that OC-iTc_REG_, in the context of skeletal homeostasis, are reactive regulators (Figure 1(b)) because they limit osteoclast activity and because they are generated by active osteoclasts. In contrast to the previous aspects of osteoimmunology, where modulation of bone homeostasis was through tonic regulators, the regulation by Tc_REG_ is by reactive regulation. Indeed, as RANKL administration induced Tc_REG_ via activation of osteoclasts, this indicates the RANKL functions both in a tonic and reactive regulatory modes.

Our *in vivo* data also indicates that active osteoclasts are needed to induce Tc_REG_, because, among other reasons, the numbers of Tc_REG_ increased in response to RANKL. To test for the induction of Tc_REG_ *in vivo*, we adoptively transferred highly purified CD8 T cells from FoxP3^eGFP^ reporter mice [70] that were depleted completely by cell sorting of all GFP and CD44 positive cells. Conversion of GFP negative to GFP positive cells was observed upon RANKL induction only in the bone marrow. This conversion from GFP negative to positive was not observed in mice pretreated with Zoledronate indicating that active osteoclasts are required for the conversion (Buchwald and Aurora, unpublished observations). These results indicate a reactive mode of regulation, consistent with the negative feedback loop motif. The negative feedback motif is also found in the immune system and serves two purposes. First it limits weak responses by acting as buffer. And second it produces strong “spikes” of activity rather than prolonged weak activity. The role in the immune system is clear: produce a burst of strong response that is of limited duration to kill a pathogen but not to damage the host. The role of such spikes of activity is less clear in the skeletal system. We suggest that as osteoid formation and mineralization by osteoblasts are relatively slow steps, the delay created by OC-iTc_REG_ could be to prevent hyperresorption by osteoclasts, while providing osteoblasts a chance to fill in the excavated bone, thus maintaining homeostasis. In this regard, the measured half-life of OC-iTc_REG_ is about 6 days. 

Osteoclasts remove bone by secreting acid and proteases into sealed compartments (lacunae) between the osteoclast and the bone. The protein and mineral products of the excavated bone are transcytosed from the lacunae and released through the secretory domain at the apical surface of the osteoclast [71]. Proteomics of the bone matrix shows that nearly 90% of the protein is type I collagen; the remaining 10% consists of type II collagen and over twenty other proteins [72]. Administering collagen (with adjuvant) initiates arthritis (CiA; [73]) by activating T cells [74], indicating that anticollagen T cells exist in the normal repertoire of rodents (and humans [75]). On the basis of these observations, we suggest that OC cross-presents neoantigens released by action of OC on the bone to convert autoreactive T cells into regulatory T cells so as to prevent autoimmunity. This notion is corroborated by our results demonstrating that adoptive transfer of OC-iTc_REG_ reduced the number effector T cells in ovariectomized mice. Our *in vitro* studies that OC-iTc_REG_ suppress the priming of naïve T-cells by dendritic cells indicate that they are tonic regulators of the immune system. 

One of the physiological situations where both the skeletal and immune systems play an important role is during pregnancy. The fetus being partially nonself (partially allogeneic due to expression of father's genome) has to be protected immunologically. One of the mechanisms of maintaining tolerance to protect the conceptus, among others (reviewed in [76]), is by increased production of regulatory T cells. The mother also increases bone mass in response to increased weight, and perhaps more importantly to store calcium for milk production during lactation. A physiologically precipitous decrease in estrogen is observed postpartum. Bone resorption increases postpartum in lactating females, to provide calcium for the rapidly growing skeleton of the neonate through milk, and due to changes in energy metabolism. It has been suggested by Pacifici that the same circuitry in response to a drop in estrogen levels that leads to increased bone turnover in lactating females is also responsible for the bone loss in postmenopausal women [77]. We use this example because it illustrates tonic, reactive, and spatiotemporal regulation of both systems. 

Why does the immune system regulate osteoclasts? We suggest two possibilities. First, regulatory T cells have evolved to suppress the immune system. As osteoclasts are derived from myeloid cells, they retain the ability to respond to immune signals. Just as cytokines produced by effector T cells activate osteoclast activity in inflammatory bone erosion diseases, the cytokines produced by Tc_REG_ suppress osteoclasts. In addition to ontogeny, a functional linkage may also exist. The bone marrow is the primary site of hematopoiesis. Stromal cells provide essential support and form a specialized sealed compartment (niche) for the hematopoietic stem cells (HSC) [78–81]. It has been documented that osteoclast activity modulates the egress of the hematopoetic precursors (HPC) from the niches [82, 83]. We hypothesize that the immune system may increase osteoclast activity through production of effector T cells. The effector T-cell secreted cytokines increase osteoclast activity during inflammation to replenish lost immune cells and thus increase circulating hematopoetic precursors (HPC). To maintain balance or restore homeostasis after inflammation, Tc_REG_ may be used to suppress osteoclast activity. For example, it is conceivable that the Tc_REG_ evolved to provide an elegant sensor for T-cell lymphopenia. A reduction in Tc_REG_ numbers may lead to an increase in bone resorption, and the subsequent increase in HPC mobilization. More studies are needed to explore the consequences of this bidirectional regulation for both the bone and autoimmune regulations and to identify the sensors that mediate this regulation.

In summary, in this review we draw parallels in the architecture of regulatory circuits that maintain homeostasis in the skeletal and the immune systems, with the intent of highlighting some of the principles of design. Three such principles in the design of such circuits are tonic, reactive (Figure 1), and spatiotemporal regulation. Such parallels are consistent (indeed expected) with the view that evolution coopts existing modules to create more specialized structures. We also review our findings with osteoclast-induced Tc_REG_ within the context of these principles of design (summarized in Figure 3).
